# Similar Cerebral Motor Plans for Real and Virtual Actions

**DOI:** 10.1371/journal.pone.0047783

**Published:** 2012-10-24

**Authors:** Chiara Bozzacchi, Maria Assunta Giusti, Sabrina Pitzalis, Donatella Spinelli, Francesco Di Russo

**Affiliations:** 1 Department of Education for Motor Activity and Sport, University of Rome “Foro Italico”, Rome, Italy; 2 Neuropsychology Centre, IRCCS Santa Lucia Foundation, Rome, Italy; 3 Department of Psychology, University of Rome “La Sapienza”, Rome, Italy; The University of Western Ontario, Canada

## Abstract

A simple movement, such as pressing a button, can acquire different meanings by producing different consequences, such as starting an elevator or switching a TV channel. We evaluated whether the brain activity preceding a simple action is modulated by the expected consequences of the action itself. To further this aim, the motor-related cortical potentials were compared during two key-press actions that were identical from the kinematics point of view but different in both meaning and consequences. In one case (*virtual grasp*), the key-press started a video clip showing a hand moving toward a cup and grasping it; in the other case, the key-press did not produce any consequence (*key-press*). A third condition (*real grasp*) was also compared, in which subjects actually grasped the cup, producing the same action presented in the video clip. Data were collected from fifteen subjects. The results showed that motor preparation for *virtual grasp* (starting 3 s before the movement onset) was different from that of the *key-press* and similar to the *real grasp* preparation–as if subjects had to grasp the cup in person. In particular, both *virtual* and *real grasp* presented a posterior parietal negativity preceding activity in motor and pre-motor areas. In summary, this finding supports the hypothesis that motor preparation is affected by the meaning of the action, even when the action is only virtual.

## Introduction

The spread of technological instruments has simplified our lives, allowing us to easily accomplish many complex actions; thus, people are used to interacting with technological instruments and controlling them with simple movements. For instance, in our daily lives, we frequently press a button to switch channels on the TV, to call an elevator, to send e-mails on a computer, or to perform an “out and out” action, i.e., while playing video games. Thus, a very simple movement, such as a key-press, can have multiple meanings and different outcomes.

So far, it is not clear whether the motor preparation of an action, such as a key-press, could vary with the additional meaning of that action, i.e., when the key-press produces a specific consequence. Alternatively, the preparation might be entirely defined by the kinematics of the movement, which, obviously, does not change with the specific result of the action.

The neural bases of action motor preparation have been widely studied using the motor related cortical potentials (MRCPs). The MRCPs are characterized by two pre-movement components: the Bereitschaft Potential (BP) and the Negative Slope (NS’). The BP is thought to be related to readiness for the forthcoming action [1], [2], as it begins well before the movement (from 1 to 3 s) and reflects early motor preparation in the supplementary motor area (SMA) and, according to more recent studies, in the superior and inferior parietal lobe [3], [4]. The NS’ has been associated with the urge to act; it starts about 500 ms before the movement and reflects activity in the pre-motor area (PMA) [5], [6].

The MRCPs literature shows that several factors related to movement are able to modulate the motor preparation. Among these factors, the complexity of the movement plays an important role. For instance, praxic movements (i.e., movements implying interaction with an object) or sequences of finger movements compared to a single finger abduction (or flexion), or the speed and precision of execution and the free movement (self-paced movement) instead of externally triggered movement, affect the onset and the amplitude of both the BP and NS’ components [3], [4], [5], [7], [8], [9], [10].

The aim of the present study was to investigate whether identical simple movements producing different consequences were supported by identical motor preparation. Our hypothesis was that the specific cognitive value of a movement (related to its consequences and its goal) was able to affect the motor preparation of the movement itself.

Support for this hypothesis comes from a previous study from our group [4] that showed how the awareness of the possibility/impossibility of achieving a specific goal affected the action preparation. In fact, when the grasping action was hindered by closing the subject’s fingers with a band, the awareness of being unable to accomplish the action modulated the BP component with respect to the real grasp condition. In addition, studies on monkeys and humans have provided evidence that during either the execution phase or observation of the execution of a movement producing different outcomes (i.e. finger flexion for grasping vs. scratching; pulling vs. pushing), regions belonging to the inferior parietal lobe (IPL) and inferior frontal gyrus (IFG) encoded for the outcome of the action rather than for its kinematic aspect [11], [12].

In the present study, we tested a very simple action by producing different effects: in one condition, the key-press had no consequences (called “*key-press*”); in another condition, the key-press triggered a video clip showing a hand grasping a cup from an egocentric point of view (called “*virtual grasp*”). As a control condition, we considered a *real grasp* of a cup, a complex movement associated with a complex motor preparation activity [4] but that shares similar cognitive aspects with the “*virtual grasp*” condition, in particular the (real or virtual) interaction between hand and object. Comparing the MRCPs associated with these three actions, we would be able to verify to what extent the goal of the action and the kinematics of the action modulated the motor planning.

It is worth noting that the present investigation expresses a different point of view with respect to virtual reality studies. In those studies, a particular brain-computer interface allows people to interact with a computer-generated environment in a naturalistic fashion (see [13]) and requires performing specific actions (such as grasping, throwing, or reaching for an object) within a simulated environment [14], [15], [16]. In the present study, we investigated a simple motor behavior (key-press), which had the power of producing a complex, simulated, and virtual action.

## Materials and Methods

### Subjects

Fifteen volunteer university students (mean age 24.7 years; SD 6.2; 9 females) participated in the study. None of the participants had a history of neurological or psychiatric illness, and all the participants were right-hand dominant, according to the Edinburgh handedness inventory [17] (LI>60; mean score 85). All subjects previously participated in our study investigating MRCPs for grasping actions [4], and they were all called back to perform the two key-press experiment investigated here.

### Ethics Statement

After a full explanation of the procedures, all subjects provided their written informed consent prior the experiment. The study and all procedures were approved by the independent the IRCSS Santa Lucia Foundation of Rome ethics committee.

### Tasks

**Figure 1:**
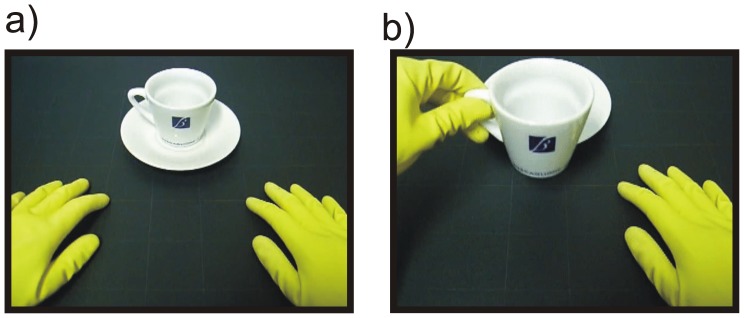
Representation of hands position at different stages. A) Starting position: the figure represents the starting image shown in both the *key*-*press* and *virtual grasp* conditions. B) In the *virtual grasp* condition, after the key press, the starting image was followed by a video clip representing the hand moving toward the cup, grasping and lifting it up (final frame). This image is also representative of the actual action performed by the participant in the *real grasp* condition.

Participants were comfortably seated on a chair in front of a table with a monitor on top and were required to perform three tasks in three separate blocks. In two blocks, subjects were required to press a key on the keyboard located on their legs (subjects were prevented from seeing their own hands). A 24-inch monitor, located in front of the subject at a distance of 35 cm, showed the static image of two hands laid on the table (the same table that was in front of the subjects) in a resting position presented from an egocentric point of view: the hands were in the bottom of the screen, the fingers pointed away from the subject, and a tea cup was located in the middle-upper part of the screen (figure 1A). The hands wore yellow gloves and a white coat, which made distinguishing whether the hands belonged to males or females impossible. Moreover, in order to facilitate identification with the character in the image, subjects were requested to wear the same yellow gloves and a white coat.

In the first task (hereafter called *key-press* task), the subjects were instructed to press the left or right button of the keyboard with the left or right index finger, according to the left or right orientation of the tea cup handle. After the key press, the image remained steady for 2 s, followed by a new image displayed with the opposite cup handle position. In a second task (hereafter called *virtual grasp* task), the setting was identical to the former, except for the fact that the key-press triggered a video showing one of the hands (according to the left/right key press, which was in turn indicated by the handle position) moving toward the tea cup, grasping and lifting it up as for drinking (see figure 1B). The duration of the video was 2 s. In both the conditions the orientation of the cup was randomized. In a third task (hereafter called *real grasp* task), the monitor was removed, and the subjects had to grasp a real tea cup located on the table at a convenient distance (35 cm from the body). Starting from a resting position with their hands laid on the table (as shown in figure 1A), the subjects extended their arms, grasped the tea cup, lifted it up as to drink from it (as in figure 1B), and then put it back on the table while returning their hand to its resting position. The action was performed with the right and the left hand alternately according to the cup handle orientation (which was switched for each new trial by the experimenter), and its duration was approximately 2 s. This third condition has already been thoroughly described in a previous study [4].

### Stimuli

The authors filmed the video clips. In the *virtual grasp* condition, in order to match the stimuli for left and right hand movements, video clips were also mirrored using video editing software (Ulead VideoStudio 9.0), and the stimuli were counterbalanced so that 50% of the videos of one hand’s movements were actually mirror movements of the other hand. We selected the first frame of the clip to create the static image of the hands in the resting position with the tea cup in the middle (figure 1A). This static image was used both in the *virtual grasp* and in the *key-press* conditions. Stimulus timing was controlled with the Presentation software (Neurobehavioral Systems, Davis, CA), triggered by the keyboard used by the subjects. The size of the hands and the cup in the static images and the video simulated the real size of the objects.

### Procedure

The tasks were executed in separate blocks. Each block included 10 runs; each run was composed of 24 trials (12 *per* hand). The *virtual grasp* condition was always performed after the *key-press* task, in order to ensure that the subjects performed the simple key-press without bias. The three conditions were performed in a block design paradigm. In particular, in order to elicit a better response, even the two key press conditions were not randomized: it was important subjects knew in advance what to expect from the key press in order to prepare the appropriate action. In this way, we could measure, if present, differences between motor preparations in the two cases.

Every action needed to be performed at a self-paced rate because we were interested in voluntary, not externally triggered, movements. The subjects were instructed to take their time before performing the task, and the interval between each action was approximately 10 s. The subjects were also trained not to count or synchronize their start either with the image onset or with the cup switching (in *grasp* condition). The subjects received online feedback when they were too fast in starting the movement and were trained to maintain, during all tasks, a stable posture and fixation on the small logo depicted in the center of the tea cup.

### ERPs Recording and Data Processing

Electrical brain activity was recorded during the tasks using a BrainVision™ 64-channels system (Brain Products GmbH, Munich, Germany) connected to an active sensor system (ActiCap™ by Brain Products GmbH, Munich, Germany), adopting the standard 10–10 system montage. The system included four electromyographic (EMG) channels with bipolar recording located at the left and right deltoids, and two channels for electrooculogram (EOG). A vertical EOG was recorded from above the left eye and a horizontal EOG from the left and right outer canthi. The left mastoid (M1) was used as initial reference electrode for all scalp channels. The signal was digitized at 250 Hz, with an amplifier band-pass from 0.001 to 60 Hz with a 50 Hz notch filter. To further reduce high frequency noise, the time-averaged MRCPs were filtered at 8 Hz. For the key-press conditions, the movement onset was triggered through the keyboard used by the subjects. In the *real grasp* condition, the EMG signal was rectified and used to identify and manually mark the first activity of the muscle (movement onset). Data analysis was conducted using BrainVision™Analyzer 1.5 (Brain Products GmbH, Munich, Germany). Data were segmented in epochs from 3500 ms prior to movement onset to 1000 ms after it. Semi-automatic computerized artifact rejection was performed prior to signal averaging in order to discard epochs with ocular or muscular contraction artifacts from further analysis. The trial recorded for each condition were 150, but on average 20% of trials were rejected. Blinks were the most frequent cause for rejection. The baseline was calculated from −3500 to −3000 ms. The time period used for statistical analysis started 3000 ms prior to movement onset and lasted until 1000 ms after movement onset.

For the statistical analysis, the mean amplitudes and onset times of the BP and NS’ and the peak of the motor potential (MP) were obtained from the MRCPs analysis on the electrodes showing an intense activity in all the three conditions and chosen because more representative. The BP onset was calculated as the first deflection that was larger than twice the absolute value of the baseline mean. Onset timing for the NS’ components were established by a visual inspection carried out by the first author and independently carried out again by the last author. The BP amplitude was calculated as the mean amplitude of the BP component (from the BP onset to the NS’ onset). Similarly, the NS’ amplitude was calculated as the mean amplitude from its onset to the MP peak latency. The MP was measured at peak amplitude, roughly corresponding to the onset of the movement. Statistical comparisons were carried out to verify significant differences between conditions of the MRCP components latency and amplitude using a 3×2 repeated-measure ANOVA with task and hand as within-subjects factors. The Bonferroni post-hoc correction was used to interpret the main effects, while the Tukey HSD post-hoc was used to interpret interaction effects. All significant effects were reported at an alpha value of.05. To visualize the voltage topography of the MRCPs components, spline-interpolated 3D maps were constructed using the Brain Electrical Source Analysis system (BESA 2000 version 5.18, MEGIS Software GmbH, Gräfelfing, Germany).

We also compared scalp topographies of different conditions by measuring statistical differences with a non-parametric randomization test as the topographic analysis of variance (TANOVA). In order to assess the TANOVA, data were average referenced and transformed to a global field power (GFP) of 1, which prevents any influence from higher activity across the scalp topographies (for more details, see [18]). Significant topographical differences were considered to occur if they were consistently present for at least 150 ms (5% of the whole epoch). This analysis provides a statistical method to determine differences between the brain networks and activation timing underlying the studied conditions.

To produce a model of the intracranial sources of the MRCP components, the Brain Electrical Source Analysis system (BESA 2000 version 5.18, MEGIS Software GmbH, Gräfelfing, Germany) was used. The spatio-temporal dipole analysis of BESA was used to estimate the orientation and time course of multiple equivalent dipolar sources seeded in known locations by calculating the scalp distribution, which was obtained for any given dipole model (forward solution). This distribution was then compared with the actual MRCPs. Interactive changes in the orientation of dipole sources led to the minimization of residual variance (RV) between the model and the observed spatio-temporal distribution of MRCPs. The position of the electrodes was digitized and averaged across subjects. The 3-D coordinates for each dipole of the BESA model were determined according to the Talairach axes and scaled according to the brain size. In these calculations, BESA used a realistic approximation of the head (which was based on the MRI of 24 subjects), and the radius was obtained from the group average (85 mm). The possibility that dipoles would interact was reduced by selecting solutions with relatively low dipole moments with the aid of an “energy” constraint (which was weighted 20% in the compound cost function as opposed to 80% for the RV). The optimal set of parameters was identified in an iterative manner by searching for a minimum in the compound cost function. Dipoles were fitted sequentially. Latency ranges for fitting were chosen (see results) to minimize overlap among successive, topographically distinct components. To minimize cross-talk and interactions between sources, dipoles that accounted for earlier portions of the waveform were left in place as additional dipoles were added. The fit of the dipole model was evaluated by measuring its RV as a percentage of the signal variance, as described by the model, and by applying residual orthogonality tests (ROT; e.g., [19]). The resulting individual time series for the dipole moments (the source waves) can also be subjected to an orthogonality test, which will be referred to as a source wave orthogonality test (SOT; [19]). All t-statistics were evaluated for significance at the 5% level.

## Results

### Waveform Analysis

**Figure 2:**
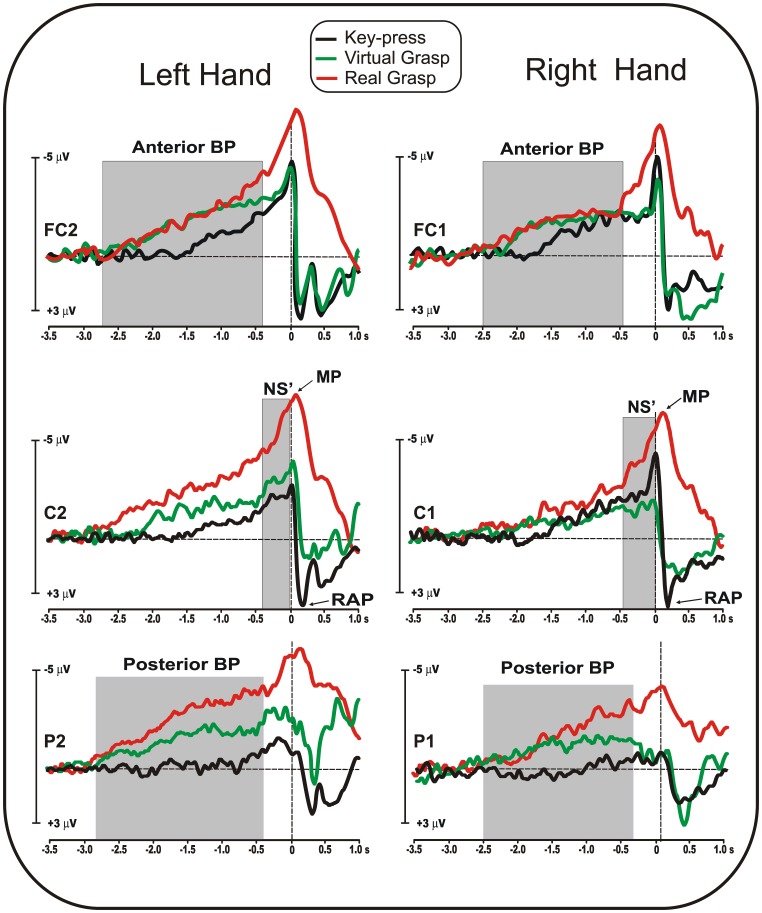
MRCPs activity from relevant electrodes for both the left and right hands in the *key-press* (black line), *virtual grasp* (green line) and *real grasp* (red line) conditions. Major MRCPs components are labeled.

Figure 2 shows the MRCP waveforms recorded at the most relevant locations (FC1/2, C1/2 and P1/2) in the three conditions (*key-press, virtual grasp* and *real grasp*).

The earliest cortical activity (the BP component) started at about 2.8 s before the movement in the *real grasp*, −2.7 s in the *virtual grasp* (both on contralateral parietal electrodes) and −1.7 s in the *key*-*press* (on FC1/2). The task effect on the BP latency was significant (F_(2,28)_ = 10.23, p<0.0005) and post-hoc comparisons showed that the *key-press* preparation started later (p<0.02) than that of the other two conditions, which did not differ from each other. Additionally, the hand effect was significant (F_(1,14)_ = 4.63, p<0.05), indicating that the BP onset was about 200 ms earlier for left- than right-hand movements. The interaction between factors was not significant.

In the *key*-*press* condition, the BP peaked on the contralateral fronto-central sites (anterior BP), whereas in the *virtual grasp,* it peaked more posteriorly on contralateral centro-parietal sites (posterior BP). For the *real grasp* condition, the BP peaked on contralateral parietal sites (posterior BP). Statistical analysis showed that the BP amplitude was affected by task (F_(2,28)_ = 10.28, p<0.0005) and hand (F_(1,14)_ = 4.76, p<0.05); the interaction was also significant (F_(2,28)_ = 3.55, p<0.05). Post-hoc comparisons showed that the BP amplitude for both hands was smaller (p<0.005) in the *key*-*press* condition than the other conditions, which did not differ. The BP amplitude for the *real grasp* condition was larger (p<0.002) for left- than right-hand movements; in contrast, this difference was not significant in *virtual grasp* and *key-press* conditions.

The NS’ onset ranged from 450 ms to 730 ms before the movement according to the task. An ANOVA showed significant task effects on the latency of the NS’ onset (F_(2,28)_ = 20.45, p<0.00001). Post-hoc comparisons showed that the NS’ for *key*-*press* was later (p<0.001) than that for the other two conditions. For *real* and *virtual grasp* conditions, the onset of the NS’ did not differ. Neither the hand factor (p = 0.81) nor the interaction (p = 0.17) was significant. The NS’ peaked in all conditions on contralateral central sites, and its amplitude was affected by task (F_(2,28)_ = 25.48, p<0.00001), but the hand factor (p = 0.07) and the interaction (p = 0.2) were not significant. Post-hoc comparisons showed that the NS’ was larger in the *real grasp* condition (p<0.0003) than in the other conditions.

The MP peaked 30–80 ms after the key-press or the *real grasp* onset, and the latency was not affected by either task or hand. Similar to the NS’, the MP was prominent in all conditions on contralateral central sites, and its amplitude was affected by task (F_(2,28)_ = 10.35, p = 0.0004). Post-hoc comparisons showed that the MP was larger in the *real grasp* condition (p<0.01) than in the other conditions, which did not differ. The effects of hand and the interaction were not significant.

After the movement, the re-afferent potential (RAP) was present in *key-press* and *virtual grasp* conditions, peaking at 160–200 ms after movement on contralateral central electrodes. This activity is strictly related to the somatosensory afferents elicited by the finger press on the key, and thus, it is very different from the activity elicited in the *real grasp* condition, wherein the movement onset was followed by the transport phase of the arm toward the cup, which was reached and touched on average 1.5 s after the movement onset. However, because the study of post-movement activities was outside the scope of the present study, which focuses on motor planning, the RAP was not further analyzed.

### Scalp Topography, TANOVA and Source Analysis

**Figure 3:**
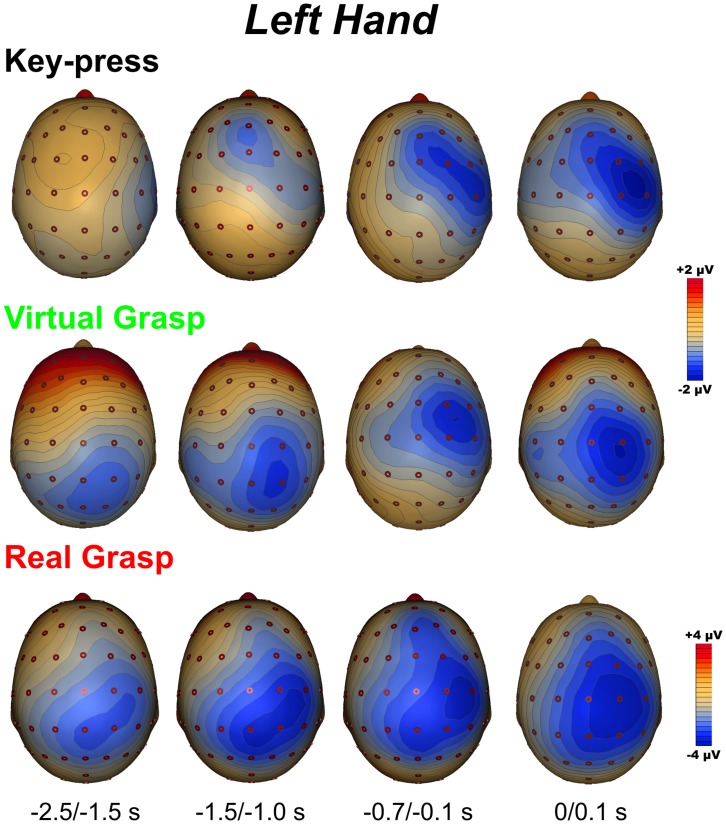
Top view of topographic 3-D voltage maps for left movement in the *key*-*press*, *virtual* and *real grasp* conditions. The four time windows shown correspond to the found MRCP components.

**Figure 4:**
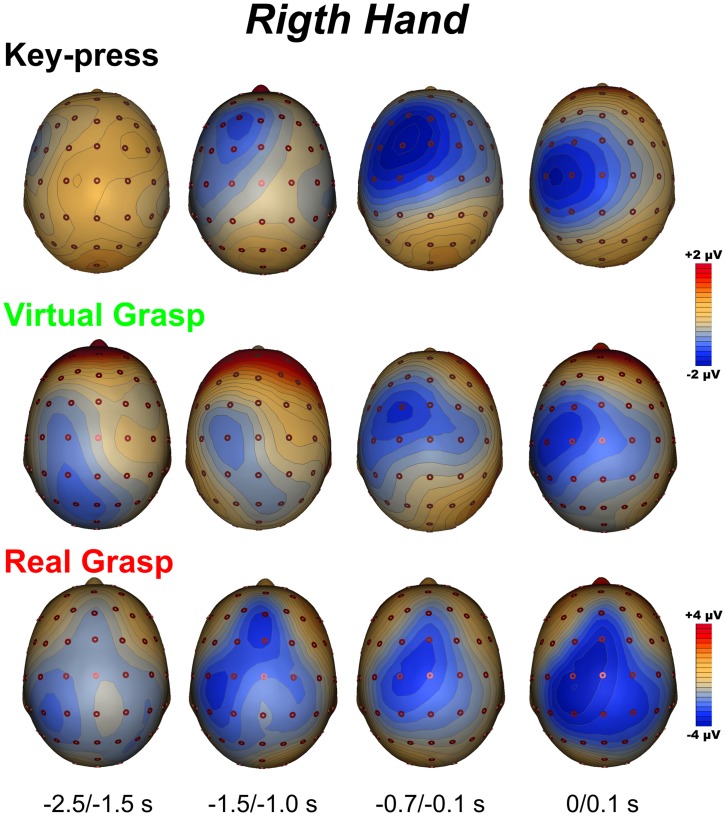
Topographic 3-D maps for right movement in the three conditions and in the four time windows.

Figures 3 and 4 show the spatial distribution of the MRCP activity in the three conditions for the left and right hand, respectively.

#### *Key-press* condition

The BP topography focused on the contralateral frontal areas, starting from −1.5 s; this activity was followed by the broader and larger distribution of the NS’ component that focused on contralateral fronto-central scalp from −0.7 s. The MP was more lateralized, focusing more posteriorly than previous components on contralateral central sites. Notably, in this condition, we did not see activity in the parietal lobes during the preparation phase.

#### *Virtual grasp* condition

The BP showed a parietal distribution (slightly contralateral) starting approximately 2.5 s before the movement. From −1500 s, the activity became wider and more anterior: the NS’ and the MP scalp topographies were similar to the *key-press* condition described above.

#### *Real grasp* condition

The BP scalp topography showed early activity on posterior bilateral parietal areas, more intense activity on the contralateral site and more widespread and earlier activity for the left hand movements. This negativity shifted anteriorly on the central sites, slightly contralateral to the hand used. The distribution of the NS’ was prominent on the contralateral fronto-central area of about 0.7 s before the MP, which focused more posteriorly on a contralateral central site.

**Figure 5:**
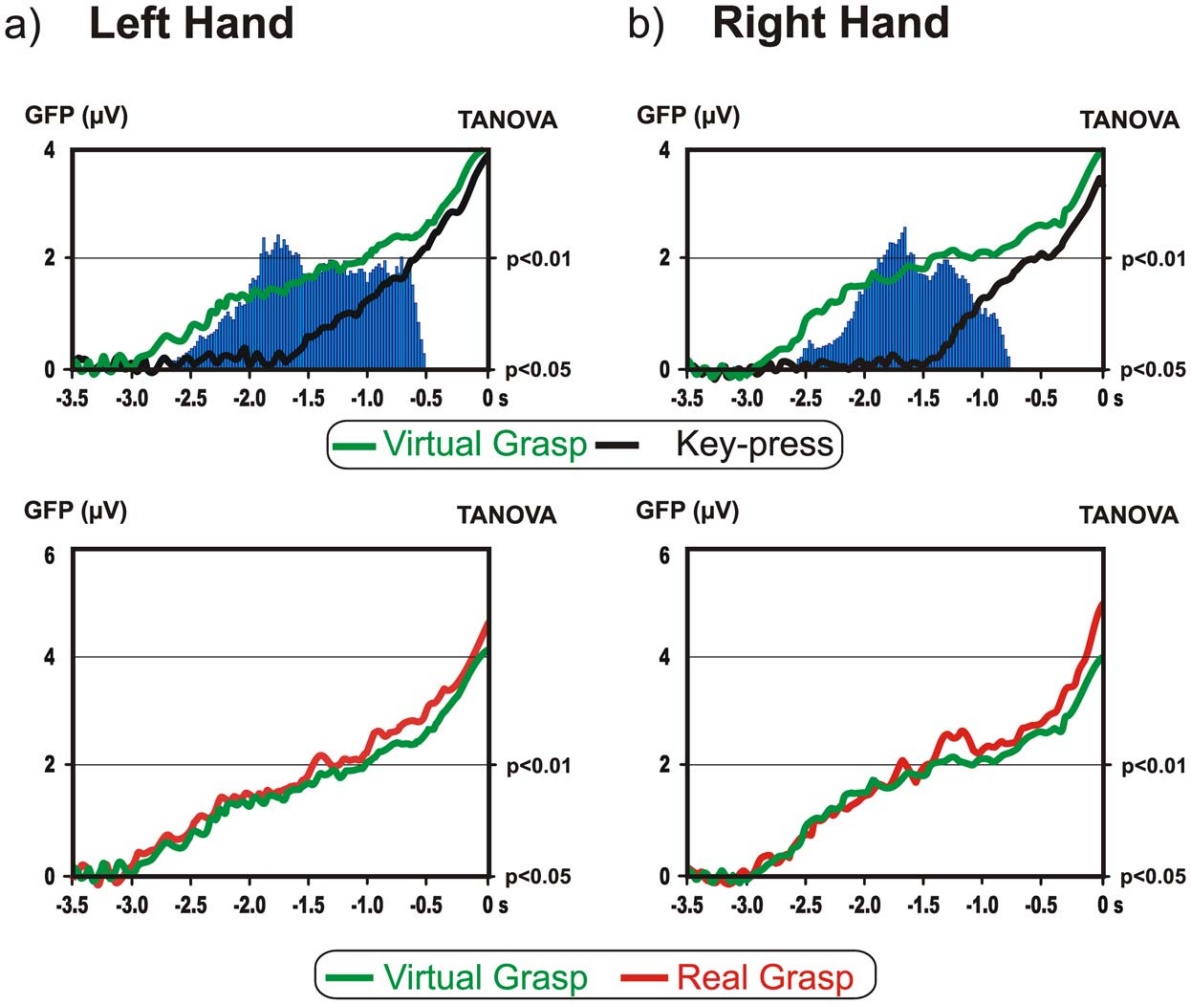
Global field power time course and TANOVA results (vertical bars) for *virtual grasp* vs. *key*-*press* conditions and for *virtual grasp* vs. *real grasp* conditions for a) left and b) right movement. The differences (vertical bars) have been plotted only if significant for at least 150 ms.

The TANOVA was carried out between *virtual grasp* and *key-press* and between *virtual* and *real grasp* (figure 5) in order to evaluate the differences and similarities of the brain networks underlying those conditions. The comparison between *virtual grasp* and *key-press* revealed that the topographies were statistically different in the interval between −2500 and −500 ms (*p*<0.05) for both left and right movements. The *virtual* and the *real grasp* conditions did not differ at a topographical level during the motor preparation period.

**Figure 6:**
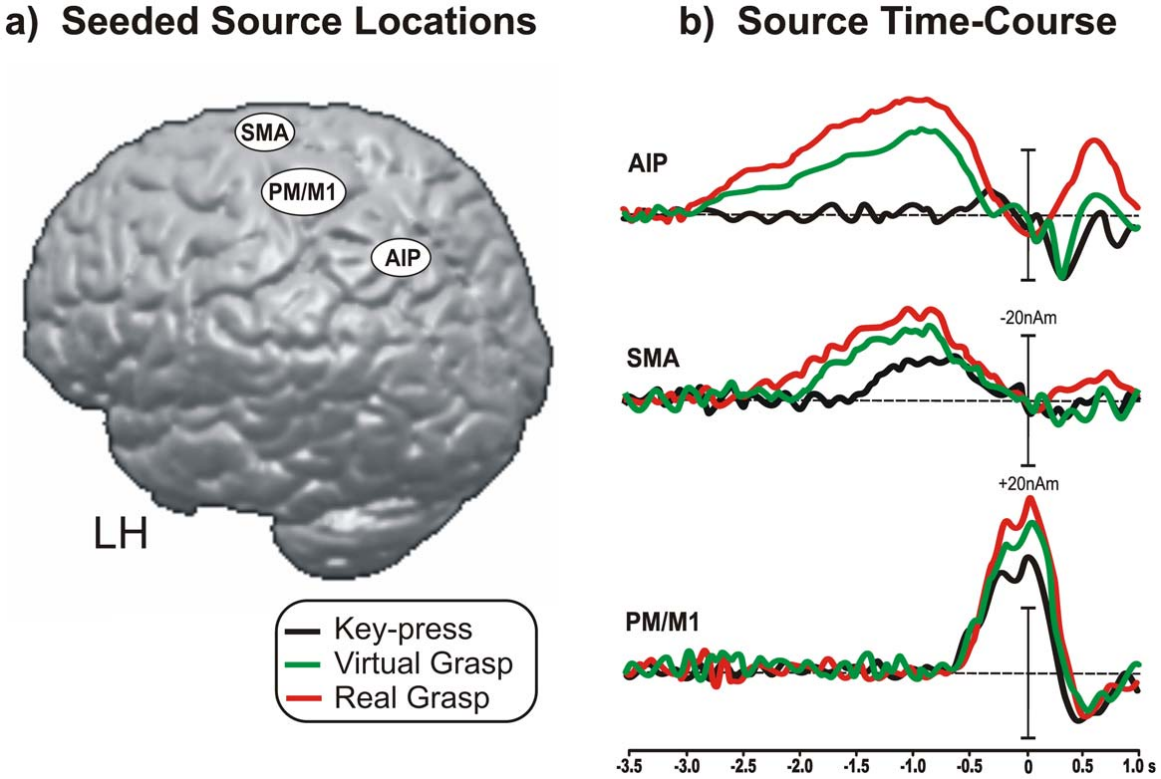
Source model of the found MRCPs components. a) Source locations seeded in the AIP, SMA, PMA/M1 and projected on a realistic model of the brain. b) Time course of each seeded cortical sources modeled separately for the three conditions and coded with different colors.

Figure 6 shows the source model of the MRCPs for the three studied conditions. The scalp topography (figures 3 and 4) shows a sequence of at least three distinctive patterns in the parietal, mid-frontal and central scalp that likely correspond to the cortical areas involved in the grasping action [20]; [21]; [22]. Thus, according to the aforementioned literature [6]; [20]; [21]; [22]; [23], we seeded the source model location to the areas corresponding to the anterior intraparietal area (AIP, Talairach coordinates −40, −50, 45), the SMA (Talairach coordinates −2, −10, 60), the PMA and M (Talairach coordinates −40, −7, 50) and the SMA (Talairach coordinates −2, −10, 60). Because of their vicinity and the low resolution of MRCPs source localization, PMA and M1 were considered as a single source (Talairach coordinates −40, 7, 50). After fixing these locations (figure 6A), we were able to calculate the time course of the activity of these areas in the three conditions. To increase the signal to noise ratio (typically low in this kind of potential), waveforms were collapsed across the left and right movements. The AIP source was fit into the interval between −2.5 and −1.5 s to account for the posterior BP. The SMA source was fit into the interval between −1.5 and −1.0 s to account for the anterior BP. The PMA/M1 source was fit into the interval between −0.7 and 0 s to account for the NS’ and MP. This sequence of intervals is the same of that used in several previous source localization studies of MRCPs (e.g. [6], [23]). Figure 6B shows the time course of the aforementioned sources showing separately (with different colors) the source waveforms of the three conditions for each seeded area. For the *key-press* condition, the AIP source was not active before the movement. The SMA source started at approximately −1.5 s before the movement, peaked at approximately −0.7 s, and became inactive at approximately −0.2 s. The PMA/M1 source started at −0.7 s, first peaked at −0.2 s (NS’ peak) and then peaked concomitantly with the movement onset (MP). For the *virtual grasp* condition, the activity in the AIP source started at approximately −2.8 s, peaked at −0.8 s and became inactive at −0.2. The SMA source started at approximately −1.9 s, peaked at approximately −0.9 s, and became inactive at the movement onset. The PMA/M1 source time course for the *virtual grasp* condition was very similar to the *key*-*press* condition, although the former condition produced larger peak amplitudes. For the *real grasp* condition, the activity in the AIP source started at approximately −3.0 s, peaked at −0.9 s and became inactive at −0.2 s. The SMA source started at approximately −2.4 s, peaked at approximately −1.0 s, and became inactive at the movement onset. The PMA/M1 source time course was very similar to the two previous conditions but had larger peak amplitude.

## Discussion

The present findings showed that motor preparation of a virtual grasp, mediated by pressing a key, was similar to that of a real grasp and was different from the preparation of a simple key-press. Although the movement in the two key-press tasks (*key-press* and *virtual grasp*) was the same, two major differences in their motor preparation were observed. The first difference concerns the BP onset latency: in the *key-press*, the BP onset was approximately 1.7 s before the movement, consistent with previous findings on simple movement preparation [24], [25]; in contrast, for the *virtual grasp*, the BP onset was more than 1 s earlier and matched the onset recorded for the *real grasp* task. The second difference concerns the cortical areas involved in motor preparation. To prepare the *key*-*press*, the anterior motor and premotor areas contralateral to the hand used accounted for all the recorded activity, as already observed in several previous studies (see 6 for review); in contrast, to prepare the *virtual grasp* task, the superior parietal areas also contributed at a very early stage, as in the case of the preparation for the *real grasp* task (called “posterior BP”; [4]). This latter result is a novel finding of the study. Thus, the present data suggest that the *virtual grasp* task and the *real grasp* task share the same preparatory cortical activities, in terms of both anatomy and timing.

Source analysis suggested that the posterior BP, for both *virtual* and *real grasp* tasks, was well accounted for by the source seeded in the anterior intra-parietal sulcus, corresponding to the typical anatomical position of the AIP area [20]. The AIP area is primarily involved in grasping action in which a transport phase of the hand prior to the grasp can be present or not [20], [26], [27]. This area would encode the goal of grasping, rather than specify details such as the trajectory of hand movement [12], [28], [29], [30]. Moreover, several fMRI studies investigating action execution and action observation have found a bilateral involvement of this area (as we found in the present study) in both modalities, reinforcing the concept that AIP function is not strictly related to the kinematics of the movement but to a broader representation of action [31]. This function might explain the very similar parietal activity recorded in the present study during preparation of the *virtual* and *real grasp* actions: the goal of the actions was indeed quite similar. Independent from the different kinematic complexities of the action performed in the two cases (which were very different), the anterior intraparietal area was the earliest activated region, and thus, it appears to be more related to the planning phase of motor preparation. According to the literature [5], [32], the motor planning represents the phase related to the general idea of the action the individual is going to perform. In that phase, a general representation of the movement and its goal are created. During the subsequent phase (motor programming), the goal is transformed into a detailed kinematic program, and finally, the execution phase follows. The present findings are in line with the literature that shows a clear differentiation between the two sub-phases: when an action involves an interaction with an object, the motor preparation includes a planning phase that takes place in the parietal areas, which are responsible for the tool affordance representation and the sensory-motor transformations required for executing movements directed at the external world. What is more appealing here is the finding that such parietal planning was also present in the *virtual grasp*, which just simulated the interaction with the object.

One could argue the possibility that the presence of the cup could have elicited an automatic response or an automatic motor behavior, as previous studies by Tucker and coll. [33] suggested. The lack of parietal activity in the preparation phase of the *key press* condition, however, does not support this hypothesis. Indeed, the cup was visible in the latter condition as well, conveying the same affordances as in the case of the *virtual grasp* task; however, in the *key-press* task, it represented only the cue to press the left or right key. The lack of consequence of the key-pressing confirmed the poorness of meaning of the *key-press* action: it was only the last step of the trial, and it did not produce any additional consequence; furthermore, the interaction with the object (the key) was minimal, reducing the task to finger flexion.

We proposed that the similar cortical preparation observed for *real grasp* and *virtual grasp* reflected the similarity of the goal/meaning of the action; however, the present finding is open to alternative interpretations. It is possible that this similarity reflects an anticipation of the visual consequences of the task; such anticipation might correspond to a mental representation or to an imagery process of the action subjects were going to perform or observe on the screen. Following this view, the present finding indicates that these representations would implicate all the motor preparation steps including the interaction with the object. While the imagery of an action may activate the same structures active in action execution [34], we would like to stress the novel aspect of the present finding represented by the fact that this activity starts well before the action initiation.

Future research should investigate the importance in the *virtual grasp* condition of the use of a video depicting a scene from an egocentric point of view. It would be interesting to understand whether the presentation of the hands from an allocentric point of view or the observation of another subject grasping, or making the cup move without interaction from the hands, produces comparable or different involvement of the parietal areas. Differences/similarities would allow us to evaluate whether the present results in the *virtual grasp* are due to a self-attribution of agency.

If our hypothesis that the parietal involvement during the preparation phase of *virtual grasp* represents the goal of the subject’s action were supported by other studies, an interesting application of them would be in the Brain Computer Interface (BCI) field. BCI is a technique that uses electroencephalography to measure and detect the brain activity and turns it into the control commands of a computer device or artificial prostheses [35], [36], [37]. So far, BCIs have been used for neuro-rehabilitation of patients presenting severe motor and muscular disorders and who require basic communication capabilities to interact with the outside world [38]. The primary purpose of BCI is to detect the user’s intent [36]. If the action’s goal plays an important role in motor preparation, it could be used to develop BCIs that are able to recognize this motor preparation pattern and anticipate the user’s commands.

Finally, it is worth underlying that several differences between *real* and *virtual grasp* conditions were present for the anterior MRCPs components related to the last stage of preparation (NS’) and the control of execution phase (MP), where the *real grasp* task produced the largest activity. The modulation of these components could be related to the amount of muscle districts (and their cortical representations) involved in the *real grasp* movement [8], [39] and affecting the motor programming phase. Indeed, no differences were present between the two key-presses, confirming that both NS’ and MP are mostly related to the kinematical and motor aspects of the action preparation. Moreover, Davare and coll. [40] showed, during grasping preparation, that activities in the ventral pre-motor cortex and the M1 were modulated by the interaction with the object to be grasped; hence, this can explain why the late components of the MRCPs, which are generated in those cortical areas, were found here enhanced in the *real grasp* condition, in which a real object interaction was actually performed.

Second, the effect of the “hand” factor deserves a comment. This effect was found for the BP amplitude of real grasp and for BP latency in the three tasks (however, less accentuate in the *key-press*). Compared to the right hand, the non-dominant left hand actions had both longer and larger amplitude, as shown in previous studies [4], [23]. Such a difference suggests that for right-handers, left-hand actions require more demanding preparation (higher mental costs in action planning) than right-handed actions. This effect might be due to asymmetric motor representation in the two hemispheres. Several studies have shown that the cortex M1 connectivity is asymmetric, being more extended in the left hemisphere, and that the movement of the right (dominant) hand only activates contralateral motor areas, whereas for the non-dominant hand, the activation is bilateral [41], [42]. Thus, the programming of left-handed movement activates both left and right preparation areas, and the resulting activity would be more intense and widespread.

In conclusion, the final effect of the action, not the kinematics of the movement, strongly influenced the early stages of the preparation phase.
